# Effects of Proprioceptive Insoles and Specific Core Training on Postural Stability for Preventing Injuries in Tennis

**DOI:** 10.3390/jfmk9010034

**Published:** 2024-02-19

**Authors:** Giuseppe Messina, Vincenzo Cristian Francavilla, Francesco Lima, Elvira Padua, Giuseppe Secolo, Innocenzo Secolo, Angelo Iovane, Maria Chiara Parisi, Donatella Di Corrado

**Affiliations:** 1 Department of Human Sciences and Promotion of the Quality of Life, San Raffaele University, 00144 Rome, Italy; giuseppe.messina@uniroma5.it (G.M.); elvira.padua@uniroma5.it (E.P.); 2 Department of Medicine and Surgery, Kore University, 94100 Enna, Italy; vincenzo.francavilla@unikore.it (V.C.F.); mariachiara.parisi@unikore.it (M.C.P.); 3 Department of Psychological, Pedagogical and Educational Sciences, Sport and Exercise Sciences Research Unit, University of Palermo, 90128 Palermo, Italy; lima.f89@gmail.com (F.L.); studiosecolo1967@libero.it (I.S.); angelo.iovane@unipa.it (A.I.); 4 Faculty of Medicine and Surgery, University Dunarea de Jos, 800402 Galati, Romania; g.secolo@hotmail.it; 5 Department of Sport Sciences, Kore University, Cittadella Universitaria, 94100 Enna, Italy

**Keywords:** balance, tennis, stability, proprioceptive insoles, injury prevention

## Abstract

Tennis is a complex sport based on unpredictability that requires adequate physical and psychological preparation to prevent injuries. The aim of this study was to investigate the effects of 8-week specific core stability training on postural stability in competitive adolescent tennis players, aged between 14 and 19 years old. Sixty-one participants were randomly allocated into two groups: experimental (*n* = 32) and control (*n* = 29) groups. The first group wore proprioceptive insoles 8 h a day and performed a detailed training 3 times a week for 8 weeks; the second group only received proprioceptive insoles to wear 8 h a day for 8 weeks. The postural stability parameters (center-of-pressure length, center-of-pressure velocity, and 95% confidence ellipse sway area) included three assessment times: baseline (T_0_), intermediate test (T_1_), post-test (T_2_), and retention test (T_3_). Data analysis showed a significant improvement in the experimental group compared with the control group, indicating a large effect size in center-of-pressure length, ellipse sway area, and center-of-pressure velocity at T_2_ and T_3_ (*p* < 0.05). In conclusion, our results suggest that a specific and detailed core stability training plays a significant role in improving balance and postural stability in young tennis players, especially in terms of preventing the risk of injury.

## 1. Introduction

Tennis is one of the most popular sports in the world. Success in tennis depends on various factors: individual talent; high physical ability; effective coaching; appropriate equipment; an understanding of the technique a player uses; and different psychological skills [1]. This sport demands a high level of physical performance regarding speed, core muscle strength, agility, flexibility, power, and dynamic balance. The game requires fast tennis shots and the ability to quickly and appropriately change movements and forms of action. Therefore, players need to react very quickly to an incoming ball, coordinate their movements, and hit the ball. It is essential that players need to flexibly adjust their body postures to deal with the game. According to many tennis experts and coaches, the serve is one of the most important and most difficult techniques in a tennis player’s technique. The results of matches between the strongest tennis players in the world of modern tennis are determined mainly by the efficiency of serve velocity. Fernandez-Fernandez et al. [2] have found a moderate relationship between core stability and tennis serve with strong core components. 

Tennis is characterized by fast, repetitive upper limb movements, while sprinting, stopping, jumping, changes of direction, and landing apply high rotational loading forces onto the lower extremities (i.e., foot, ankle, and knee). Furthermore, tennis, unlike many other sports, does not have time limits on matches. This can result in matches lasting less than one hour or as long as five hours (in five-set matches). Due to the high stresses placed on the body, tennis players are at increased risk of many injuries (chronic and acute). In tennis, the incidence rate is about 0.05–2.9 injuries per player per year. The lower extremity is the most frequently injured region (range 40–65%), followed by the upper extremity (range 25–45%) and the head/trunk (range 8–20%). The shoulder joint is the most frequently injured site of the upper body. Tennis elbow and wrist tendinopathy are also common injuries, since tennis players impart a lot of rotation and speed to their strokes. The lower leg is the most frequently injured site of the lower body, with ankle sprains and foot injuries (e.g., Achilles tendon injuries, metatarsal fractures, and midfoot sprains) as the most common injuries [3]. Foot and ankle injuries may be prevented using proper bracing and shoe wear, as well as stretching exercises before the game [4].

In this context, knowledge of the biomechanical patterns of technique execution and accurate coordination abilities are crucial to successfully performing a variety of motor skills and preventing injuries [5]. Postural stability is the ability to maintain an upright position in a specific spatial orientation or to regain equilibrium after external dynamic perturbations, attempting to keep the center of gravity within the base of support. Furthermore, it provides the primary foundation for mobility and is a decisive factor in preventing falls and resulting injuries. Preserving body equilibrium depends on proprioceptive information resulting from three areas: the sole of the foot, the cervical spine, and the sacroiliac joint. The foot is the foundation of the lower kinetic chain [6]. In the ideal postural control mechanism, the foot plays an important role in potential modifications of foot shape, foot posture, and bone alignment, as it is a sensitive region to both tactile and vibration stimuli [7]. Specifically, it has been observed that the larger midfoot contact area found on the dominant side increases the number of receptors responsible for the spatial orientation of the body’s center of mass in contact with the ground [8]. In this regard, foot type is primarily related to the foot arch difference, which is subdivided into pes cavus (highly arched and associated with extreme hindfoot varus); pes rectus (normal); and pes planus (flat arched and associated with hindfoot valgus and excessive forefoot varus) [9,10]. The term “kinetic chain” describes the synchronous use of selective muscle groups, segmental spins, and coordinated lower extremity muscle activation that conducts the lower body force by the core to the upper body and externally through the racket into the ball. Core muscles are crucial for stabilizing the spine and reinforcing the trunk during upper extremity movements and maximizing balance in the lower extremity movements, especially in the early stages of motor development. In tennis performance, Kovacs et al. [11], have shown that decreased muscle strength and postural control lead to a diminished speed and instability, as well as an increased risk of sports injuries. In addition, core stabilization is essential for improving athletic performance, leg balance, and trunk strength, and for preventing various sports injuries [12,13,14,15]. At this point, it is possible to affirm that tennis is a complex and intermittent sport that requires an accurate combination of the major physiological variables. At the same time, it is necessary to take into account the individual approach to a specific person—the age criterion, body composition, degree of stress, and experience. For example, body composition is an essential aspect of the overall physical condition of athletes, and its assessment may aid in optimizing competitive performance [16,17].

Based on the current literature, the aim of this study was to investigate the effects of 8-week specific core stability training on postural stability in competitive adolescent tennis players. We hypothesized that this approach, consisting on one side of the use of a tailored proprioceptive insole and on the other side of specific core stability training, may induce improvements in balance and postural stability and, consequently, prevent the risk of injury.

## 2. Materials and Methods

### 2.1. Participants

**Figure 1:**
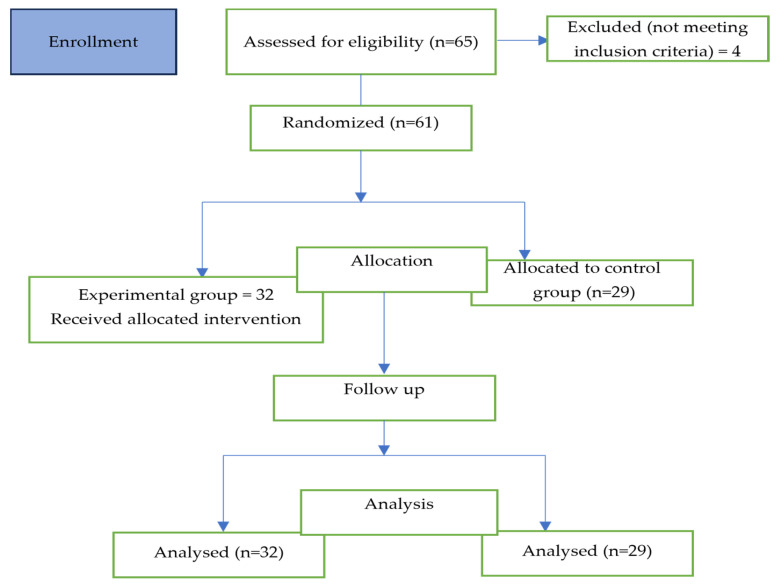
Flowchart of the study design.

A total of 65 participants were recruited to participate in the research. The inclusion criteria were as follows: absence of motor or neurological deficits, no trauma, no pain, no orthopedic injury, or vestibular damage. The exclusion criteria were those currently participating in a stability exercise protocol. Four participants receiving physical therapy were excluded. Sixty-one male competitive tennis athletes, aged between 14 and 19 years old (M_age_ = 16.1 years, SD = 1.60 years), were randomly assigned to experimental (*n* = 32) and control (*n* = 29) groups, using the toss of a coin. The flow diagram is displayed in Figure 1. The athletes were all members of sports clubs and had a minimum of 5 years of practical experience in the sport. The training schedule usually consisted of 2/3 h (2.4 ± 0.46) per day (five to six weekly workouts). Before the beginning of the study, each participant received a full explanation of the study procedures and signed a written consent form to participate. Confidentiality of the responses was also assured. For athletes under 18 years of age, a consent form was signed by their parents.

### 2.2. Procedures

All participants were tested four times at pre-determined intervals: one day prior to the first day of training, baseline was conducted (T_0_); at the end of the fourth week of the intervention (T_1_); at the end of the eighth week of the intervention (T_2_); and two weeks after training was terminated, a retention test was performed (T_3_). Yaggie et al. [18], indicated that a 2-week cessation of training can be related to a significant decrease in training effects. 

At each test, the participants were subjected to a postural stability assessment using a baropodometric platform. The assessment took place in the same laboratory with analogous conditions (room temperature 21 °C, electric illumination, time of day, etc.) by the same researchers. Measurements were conducted far away from competitions in an effort to minimize external distractions. The experimental group wore proprioceptive insoles for 8 h a day and performed a detailed training 3 times a week for 8 weeks; the control group only received proprioceptive insoles to wear for 8 h a day for 8 weeks. After the 4th full week of intervention, the study participants were asked to suspend training for 5 days and then restart for the definitive 4th full week of intervention. 

The definitive protocol lasted 65 days, of which 60 were for the intervention and 5 were rest days between the two interventions. Two weeks after training was terminated, a retention test was performed to determine whether players experienced a positive effect.

The procedures were conducted according to the ethical principles stated in the Declaration of Helsinki and were performed with university ethics approval. The study design was approved by the Departmental Research Committee (approval number: 280/2022/MEDF-02/11; approval date: 27 March 2015).

### 2.3. Intervention

#### 2.3.1. Core Stability Training

According to the basic guidelines of general training protocols [19], a specific core stability training program was exclusively created for this study aiming to increase joint range and muscle extensibility, enhance muscle strength, and stabilize the ankle and foot joints (Table 1). 

#### 2.3.2. Proprioceptive Insole

The tailor-made proprioceptive foot plantar was a complex medical device created “ad hoc” for the patient, aiming to stimulate and integrate the sensory signals from various mechanoreceptors, thereby determining a more stable body position. In a semi-sitting position, the insole was created by taking a morphological imprint of the foot on carbon paper. The base was created using a Relyon Inc. (Busan, Republic of Korea) base insole, an eco-leather cover, and corrections in reinforced felt from 1 to 3 mm. Depending on the degree of pronation or supination, raises with proprioceptive push were positioned. 

### 2.4. Measurements

#### Baropodometric Platform

The postural stability assessment was performed using the FreeMed^®^ baropodometric platform and FreeStep^®^ software 2.0 (Sensor Medica^®^; Guidonia Montecelio, 00012 Rome, Italy), with the following specific features: platform surface 440 × 620 mm, with an active surface of 400 × 400 mm and 8 mm thickness. The system samples real-time postural sway at 55 Hz. The sensors, coated with 24 K gold, guarantee repeatability and reliability of the instrument. According to the Romberg test, each athlete was in a standing position for 51.2 s, with feet placed side by side forming an angle of 30°, and both heels 4 cm apart. The examination was conducted with open eyes in total silence and while wearing comfortable clothes. The parameters of postural stability measurement were as follows: center-of-pressure length (L_CoP_—mm); 95% confidence ellipse sway area (ESA_95%_—mm^2^); and center-of-pressure velocity (V_CoP_—mm/s). Length represents the sum of postural sway in the anteroposterior and mediolateral directions, ellipse sway area represents the area of all centers of pressure, and average velocity denotes the total distance traveled by the center of pressure over time [20]. An increase in these variables represents a decreased ability to control posture; conversely, a decrease represents an improvement.

### 2.5. Statistical Analysis 

This study was designed to demonstrate an improvement in postural stability assessment after specific core stability training. The normality of data distribution was tested using the Shapiro–Wilk test. Descriptive statistics were used to calculate the mean and standard deviation (SD). Independent *t*-tests were conducted to identify statistically significant differences between the two groups at the level of postural parameters. A paired *t*-test was conducted to detect within-group differences for each assessment during sequential testing. The effect size calculation (Cohen’s d) was used for each analysis with the eta-squared statistic (η^2^) to evaluate the practical significance of findings, where d = 0.2 and η^2^ = 0.01 were considered a “small” effect size, d = 0.5 and η^2^ = 0.06 represented a “medium” effect size, and d = 0.8 and η^2^ = 0.14 represented a “large” effect size [21]. All statistical analyses were processed using SPSS version 27 (SPSS Inc., Chicago, IL, USA) and presented as mean ± SD (significance level: *p* ≤ 0.05).

## 3. Results

A total of 61 players participated in the study and were allocated to experimental (*n* = 32) and control (*n* = 29) groups. Anthropometric characteristics of the participants are illustrated in Table 2. According to the World Health Organization, a BMI of 18.5–24.9 is considered normal; a BMI less than 18.5 is considered underweight. Height and body weight measurements were performed with the athlete in a standing position, barefoot, and wearing comfortable clothing. Height was measured using a digital stationary stadiometer (seca 264, Seca, Birmingham, UK). Body weight was evaluated using a body composition analyzer (seca mBCA 515, Seca, Birmingham, UK). No adverse effects or health problems occurred during testing or training sessions.

Independent *t*-tests were conducted to identify statistically significant differences between the two groups at the level of postural parameters. Comparisons of means and standard deviations for each variable are presented in Table 3. 

A paired *t*-test was conducted to detect within-group differences for each assessment during sequential testing (baseline T_0_; after 4 weeks T_1_; after 8 weeks T_2_; and a retention test after 2 weeks T_3_), at the level of parameters considered to measure postural stability (Table 4). First, there were no significant differences in baseline characteristics between groups. In T_1_, no significant changes in the examined parameters of postural stability were found. Our data showed a significant increase in the experimental group compared with the control group, indicating a large effect size in center-of-pressure length at T_2_: F_(1,59)_ = 0.958, *p* = 0.04; and T_3_: F_(1,59)_ = 0.947, *p* = 0.04. The results were also replicated in the 95% confidence ellipse sway area at T_2_: F_(1,59)_ = 8.091, *p* = 0.01; and T_3_: F_(1,59)_ = 7.971, *p* = 0.01. The eta square statistic indicated a large effect size in center-of-pressure velocity at T_2_: F_(1,59)_ = 0.868, *p* = 0.001; and T_3_: F_(1,59)_ = 0.038, *p* = 0.03. 

**Figure 2:**
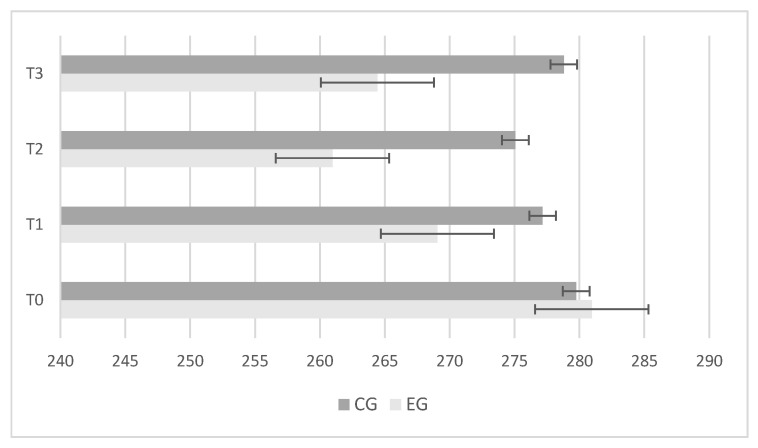
Differences between control (CG) and experimental (EG) groups in “Center-of-Pressure Length” results during baseline (T_0_); after 4 weeks (T_1_); after 8 weeks (T_2_); retention test after 2 weeks (T_3_). Error bars represent standard error of the mean.

**Figure 3:**
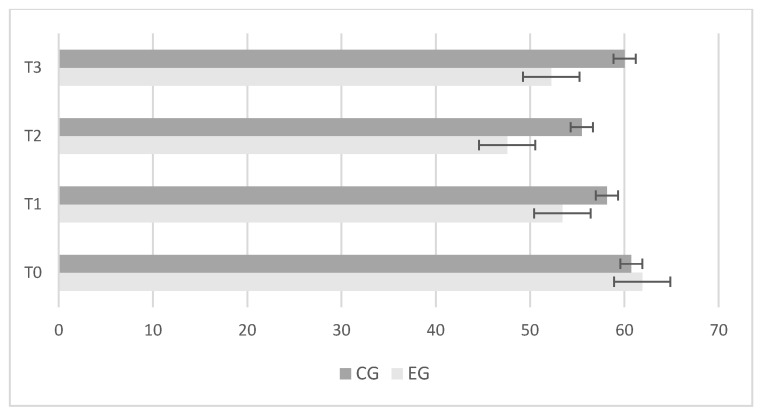
Differences between control (CG) and experimental (EG) groups in “Ellipse Sway Area” results during baseline (T_0_); after 4 weeks (T_1_); after 8 weeks (T_2_); retention test after 2 weeks (T_3_). Error bars represent standard error of the mean.

**Figure 4:**
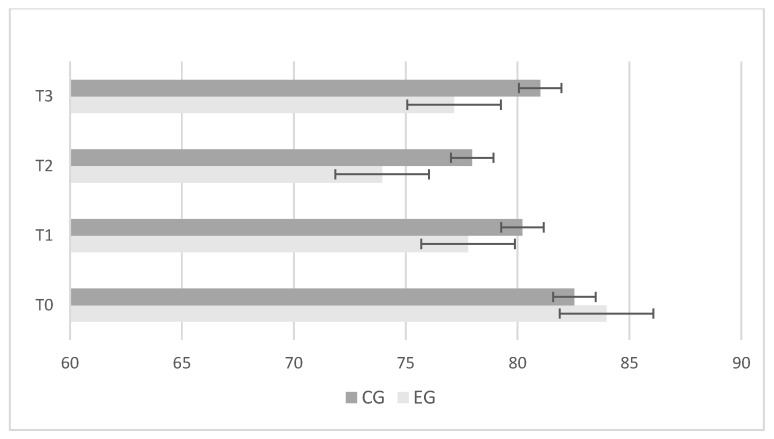
Differences between control (CG) and experimental (EG) groups in “Center-of-Pressure Velocity” results during baseline (T_0_); after 4 weeks (T_1_); after 8 weeks (T_2_); retention test after 2 weeks (T_3_). Error bars represent standard error of the mean.

The experimental group showed greater and significant improvements in center-of-pressure length (Figure 2); ellipse sway area (Figure 3); and center-of-pressure velocity (Figure 4) between T_2_ and T_3_, while there was no change in the control group. 

## 4. Discussion

The repetitive demands, and motion affect and stress on the upper and lower extremities of tennis on elite players’ bodies and can lead to characteristic injury patterns and musculoskeletal adaptations. To combat the effects of this continuous loading, preventive approaches include extensive core stability training [22].

The aim of this study was to investigate the effects of 8-week specific core stability training on postural stability in competitive adolescent tennis players. We hypothesized that this approach, consisting of the use of a tailored proprioceptive insole on one side, and specific core stability training on the other side, may induce improvements in balance and postural stability and, consequently, prevent the risk of injury. Our hypotheses have been supported by the obtained results, showing positive and foremost effects in the experimental group of young tennis players after 8 weeks of intervention. These results are in line with previous reports documenting foremost and positive effects after a specific core training program. Xiao et al. [23], showed a significant increase in physical performance in the experimental group, compared with controls, after 6 weeks of core training. Fernandez et al. [24] and Majewska et al. [25] obtained the same results after 6 weeks of specific core training and prevention exercises in a group of young, elite tennis players. Wang et al. [26] confirmed that 9 weeks of core training positively improved tennis players’ core strength. In the present study, statistical analysis showed a significantly positive effect of the entire specific protocol (training plus use of insoles) on the performance of the experimental group when, in T_2_ and T_3_ assessments, we recorded significant differences in each parameter of postural stability, indicating that the acquired ability to control posture was retained following 2 weeks of inactivity. Specifically, we observed a great improvement in the sum of postural sway in the anteroposterior and mediolateral directions, the area of all centers of pressure, and the total distance traveled by the center of pressure over time. In line with our results, Bashir et al. [27], indicated a statistically significant effect of core training on stability in junior tennis players. Furthermore, this relationship potentially may have a significant and positive impact on preventing and reducing injury risk [28,29,30]. In the sporting environment, therefore, core stability and core strength are essential for controlling the position and motion of the trunk, and for performance enhancement. 

When the body posture is unfit and the muscle strength is weak, the human body effects various compensations to re-establish homeostasis [31]. Consequently, it is important that any trunk-stabilizing muscle weakness is recognized and addressed, as this significantly increases an individual’s risk of muscle and joint injuries. 

The analysis of the control group, which only received proprioceptive insoles to wear for 8 h a day for 8 weeks, showed that there were no significant differences (*p* > 0.05). By not performing a consistent core training program, the unsatisfactory score may be attributed to poor sensory integration (reduced connection between nerves and muscles) or a larger area of center of pressure excursion, which significantly affected the area score compared to the experimental group [32]. Previous research has confirmed that core training can enable athletes to achieve higher level of postural stability, maintaining a dynamic integration of internal and external forces and improving lower body movements. Notably, during multidirectional tasks and constant changes of direction, tennis players require core stability to resist direct forces on the spine. In summary, the greater the strength of the core area, the higher the body’s stability and accuracy of technical movements during competitions [13]. 

We suppose that the significant differences and large effect sizes in the performance of the postural stability tests of the experimental group are attributable to the entire protocol composed of specific exercises to improve core muscles with the use of proprioceptive insoles. Our results provide insight into the training strategy for core stabilization in tennis players. Moreover, this specific training protocol could be very effective in obtaining relevant results and preventing the risk of injury. In fact, the athletes subsequently participated in several competitions without sustaining any injuries.

There are some limitations of this study: (a) the short duration of the follow-up; (b) no influence on the repeatability of the objective evaluation of tests used is shown. Once it has been demonstrated that this specific protocol is successful in providing improved postural stability, the information gained from future studies could then be used to develop an effective fall and injury prevention program in tennis, also involving longer follow up. Furthermore, our study was performed in a specific group of elite junior tennis players. Consequently, future research should include different age groups and ability levels.

## 5. Conclusions

Higher level of postural stability may benefit activities of daily living by providing a foundation for greater force production in the upper and lower extremities. The results of our study demonstrated the positive impact of an eight-week core stability exercise program on the quality of fundamental movement patterns as well as core stability test results in a group of elite junior tennis players. Programs for coaches and physiotherapists should constantly integrate specific protocols as a fundamental element of an athlete’s good performance. 

## Figures and Tables

**Table 1:** Core Stability Training.

The exercises have been divided into three main parts: WARM-UP Myofascial massage with roller and ball (Sets: 1—Reps: 30 s per limb) Phase 1: starting position: Place the roller or ball under the soleus-calf muscles with the arms resting on the ground and slide back and forth over the ball/roller. Phase 2: starting position: Place the roller or ball under the hamstrings with your arms on the ground and slide back and forth over the ball/roller. Phase 3: starting position: Place the roller or ball under the lateral thigh muscles. Find the balance position by placing the elbows on the ground and resting the foot of the leg opposite the one that is working on the ground. Perform a slide from this position. Phase 4: starting position: Sit on a chair and roll the ball along the sole of your foot. Proprioception (Series: 2—Reps: 10) Phase 1: In an upright position, place two proprioceptive half spheres under the plantar region, one under the region between the heel and the isthmus, the other between the isthmus and the forefoot. The contralateral lower limb is suspended. Phase 2: Maintain the position by checking the position of the ankle joint.
2. MUSCLE STRENGTHENING Strengthening exercise of the peroneal muscles (Sets: 3—Reps: 15) In the upright position, carry out an eversion movement (external rotation of the foot) against muscular resistance. Posterior tibial strengthening exercise (Sets: 3—Reps: 15) Sitting on a chair, place the elastic around the sole of the foot, with the only point of contact with the ground at the heel. Make a reverse movement (turning the foot inward). Posterior tibial strengthening exercise (Sets: 3—Reps: 15) Sitting on the ground, flex one leg while extending the other, and overcome the resistance of the elastic by performing flexion-extension of the sole of the foot. Anterior tibial strengthening exercise (Sets: 3—Reps: 15) Sitting on the ground, flex one leg while extending the other, and overcome the resistance of an elastic by performing flexion-extensions of the back of the foot. Toe-toe and lateral foot walking (Sets: 3—Reps: 60 s each) Walk on your toes with your knees extended and locked for 1 min. Walk on your heels with your knees extended and locked for about 1 min. Walk on the outside of your feet without resting the soles of your feet on the ground. Mobilization of the ankle joint (Sets: 2—Reps: 5) With feet slightly apart, place the front foot approximately 15–20 cm from the wall with legs straight. From this position, sit down, soliciting the flexion-extension of the ankle joint. The heel must remain close to the floor.
3. STRETCHING Elongation of the posterior tibialis (Sets: 2—Reps: 30 s) Phase 1: starting position: Stand upright facing a wall with your hands supported and your feet parallel. Slowly bend your left leg while abducting your right leg and slide back until your heel can maintain contact with the floor. As you exhale, rotate your pelvis, bringing your right hip forward and to the side. Breathe deeply and hold the position until you feel the maximum stretch. Elongation of the anterior tibialis (Sets: 2—Reps: 30 s) Phase 1: starting position: Kneel on your heels with the backs of your feet on the ground. Hold the position while breathing. Lengthening of the peronealis (Sets: 2—Reps: 30 s) Phase 1: starting position: Stand upright with your heels 60 cm apart and your lower limbs completely rotated (toes pointing inward). As you exhale, gradually bend your knees, bringing them closer together. Maintain the position with your pelvis in retroversion and your torso erect while breathing. Stretching of the flexors of the fingers (Sets: 2—Reps: 30 s) Phase 1: starting position: Squat in front of a wall with your hands resting on it. Gradually lower your knees until they just touch the floor. Breathe deeply and hold the position.

**Table 2:** Tennis athletes’ anthropometric characteristics. Mean ± s (range).

Groups	Age (years)	BMI (kg/m^2^)	Weight (kg)	Height (cm)
Experimental Group	15.9 ± 1.64 (14–19)	21.6 ± 1.12 (19.4–23.4)	60.8 ± 2.16 (57.0–65.0)	169 ± 2.19 (163–173)
Control Group	16.2 ± 1.57 (14–19)	21.3 ± 1.03 (19.2–23.4)	61.6 ± 2.31 (58.0–66.0)	169 ± 3.78 (162–174)
η^2^	0.23	0.17	0.21	0.19
*p*	0.85	0.61	0.73	0.67

Notes: Statistically, no differences were found between these groups with respect to age, body mass, weight, or height.

**Table 3:** Mean (±SD) for postural parameters across session and group.

Postural Parameters	Group	T_0_	T_1_	T_2_	T_3_
L_CoP_ (mm)	EG	281 ± 28.4	269 ± 28.2	260 ± 27.6 *	265 ± 27.8 *
	CG	280 ± 24.7	277 ± 24.9	275 ± 24.9	279 ± 25.4
ESA_95%_ (mm^2^)	EG	62 ± 9.4	54 ± 9.6	48 ± 8.9 *	52 ± 9.1 *
	CG	61 ± 15.6	59 ± 15.5	55 ± 15.2	60 ± 15.7
V_CoP_ (mm/s)	EG	84 ± 6.1	78 ± 6.7	74 ± 6.5 **	77 ± 7.6 *
	CG	83 ± 4.7	80 ± 4.4	78 ± 4.6	81 ± 5.9

Notes: * *p* < 0.05, ** *p* < 0.01. L_CoP_ (mm) = center-of-pressure length; ESA_95%_ (mm^2^) = 95% confidence ellipse sway area; V_CoP_ (mm/s) = center-of-pressure velocity; EG = experimental group; CG = control group; T_0_ = baseline, T_1_ = after 4 weeks; T_2_ = after 8 weeks; T_3_ = retention test after 2 weeks.

**Table 4:** Comparison of postural parameters.

Postural Parameters		t	*p*	Mean Difference (95%CI)	η^2^	Cohen’s d
L_CoP_ (mm)	T_0_	0.17	0.86	1.20 (−12.5~14.9)	0.09	0.04
	T_1_	−1.18	0.24	−8.10 (−21.8~5.7)	0.16	−0.30
	T_2_	−2.08	0.04 *	−14.10 (−27.7~−0.5)	0.54	−0.54
	T_3_	−2.09	0.04 *	−14.36 (−28~−0.7)	0.57	−0.54
ESA_95%_ (mm^2^)	T_0_	0.356	0.72	1.16 (−5.3~7.7)	0.07	0.09
	T_1_	−1.44	0.15	−4.72 (−11.2~−1.8)	0.11	−0.37
	T	−2.50	0.01 *	−7.92 (−14.3~−1.6)	0.49	−0.65
	T_3_	−2.40	0.01 *	−7.79 (−14.2~−1.31	0.53	−0.61
V_CoP_ (mm/s)	T_0_	1.02	0.31	1.44 (−1.4~4.2)	0.21	0.26
	T_1_	−1.66	0.10	−2.43 (−5.3~0.49)	0.19	−0.42
	T_2_	−2.82	0.001 *	−4.10 (−7.0~−1.2)	0.77	−0.72
	T_3_	−2.21	0.03 *	−3.87 (−7.3~−0.37)	0.69	−0.57

Notes: * *p* < 0.05. L_CoP_ (mm) = center-of-pressure length; ESA_95%_ (mm^2^) = 95% confidence ellipse sway area; V_CoP_ (mm/s) = center-of pressure velocity; T_0_ = baseline; T_1_ = after 4 weeks; T_2_ = after 8 weeks; T_3_ = retention test after 2 weeks.

## Data Availability

The data that support the findings of this study are available from the corresponding author (D.D.C.), upon reasonable request.
